# Study on environmental behaviour of fluopyram in different banana planting soil

**DOI:** 10.1038/s41598-021-91460-4

**Published:** 2021-07-28

**Authors:** Jia Zhou, Shuilian Liang, Yuanyuan Cui, Yu Rong, Jia Song, Daizhu Lv

**Affiliations:** 1 Analysis and Testing Center, Chinese Academy of Tropical Agricultural Sciences, No. 4 Xueyuan Road, Haikou, 571101 China; 2 College of Plant Science and Technology, Huazhong Agricultural University, No. 1 Shizishan Street, Wuhan, 430072 China; 3 College of Resources and Environment, Huazhong Agricultural University, No. 1 Shizishan Street, Wuhan, 430072 China

**Keywords:** Ecology, Environmental sciences

## Abstract

Fluopyram is commonly used to control banana leaf spot, anthracnose, and scab in tropical agricultural areas. To explore its behaviour in tropical agricultural environments, dissipation, adsorption, and leaching behaviours of fluopyram in three typical banana planting soils were studied. Also, its dissipation and migration capabilities in different regions and different soil types were evaluated. The results showed that the dissipation of fluopyram was in accordance with the first-order kinetic equation in the three banana soils, but the degradation rates were quite different. The degradation half-lives in the Hainan latosol, Yunnan sandy soil, and Fujian Plain alluvial soil were 46.21 days, 36.48 days and 57.76 days, respectively. Fluopyram also exhibited high adsorption and low leachability in the three soils. The Fujian Plain alluvial soil had the highest adsorption capacity for fluopyram, while fluopyram had the low leachability in the Yunnan sandy soil.

## Introduction

In modern agriculture, pesticides are essential for achieving high crop yields. However, with the increasing use of pesticides, some of them may be retained in the surface soil during the application process. They can also infiltrate into surface and groundwater through absolute input, rivers, atmospheric deposition, and other ways^1,2^, exacerbating the scales of environmental pollution. The diffusion of pesticides in the environment depends on their behaviour in the environment. Thus, it is crucial to understand the dissipation and migration behaviour of various pesticides in the soil when pesticide pollution is studied. Due to the use of a wide variety of pesticides, other metabolites may be produced in the environment. Therefore, studying the environmental behaviour of pesticides under natural conditions requires extensive records of numerical data. Such data can be used in research for evaluating the impact of pesticides and their metabolites on the environment. Soil is an important environmental medium, and the impact of pollutants on soil has become an important environmental issue.

**Figure 1:**
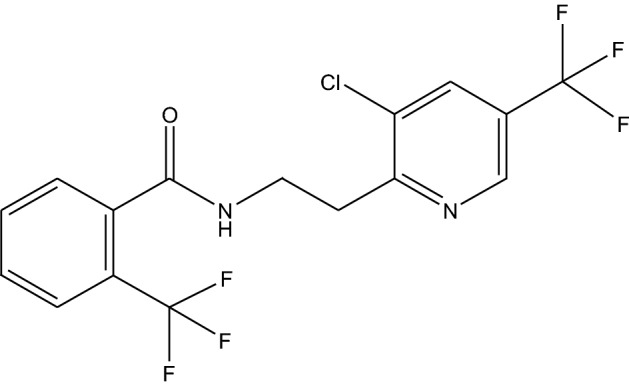
The structure of fluopyram.

Fluopyram (Fig. 1), *N*-(2-[3-chloro-5-(trifluoromethyl)-2-pyridinyl] ethyl)-2-(trifluoromethyl) benzamide, is a new generation fungicide developed by Bayer Company, Germany^3,4^. It is widely used as a broad-spectrum nematicide, seed treatment agent, preservative for the storage of agricultural products, and for several other applications. Fluopyram is a succinate dehydrogenase inhibitor (SDHI) fungicide that has a unique action site. It selectively inhibits complex II or succinate coenzyme Q reductase in the electron transport chain of mitochondrial respiration and interferes with its respiration^5,6^. Bananas are often threatened by fungal/nematode diseases during the plantation process and fluopyram can be used to control such diseases including banana leaf spot^7^, anthracnose^8^, and scab^9^. It can be applied at the early stages of the disease to achieve a good control effect. Fluopyram is also the first SDHI nematicide with low toxicity to the environment and users. The toxicity test of fluopyram to Meloidogyne incognita and *Rotylenchulus reniformis* has disclosed that low concentrations of fluopyram can effectively reduce the ability of the two nematodes to infect tomato roots, and it had a good sustained effect^10^. As fluopyram exhibits multiple effects, it is being increasingly used in banana growing areas. The use of fluopyram substantially increases the possibility of soil contamination by the pesticide in the main banana production areas.

The assessment of fluopyram residues in crops and soil has been described in previous studies. Patil^11^ sprayed pomegranate with a recommended dose and a double dose in the field to study the residual amount and dissipation behaviour of fluopyram in pomegranate and the planting soil. Dong and Hu^12^ tested the final residual amounts of fluopyram and tebuconazole in watermelon and the soil. They proved that the two pesticides were safe to use for watermelon cultivation. Wei^13^ examined the dissipation kinetics of fluopyram in typical fruits and vegetables (tomatoes, cucumbers and peppers) in the greenhouse environment. According to the hazard quotients (HQs), the consumption of fruits and vegetables, 7 days after the application of fluopyram, is seemingly safe for human health. However, each study has only evaluated the residue and digestion dynamics of fluopyram in agricultural products and soil, and none of them have thoroughly studied the adsorption and migration characteristics of fluopyram in soil (especially in tropical zones with high temperature, humidity, and rainfall). Studying fluopyram’s dissipation, adsorption and migration behaviour in tropical soil environments is urgently required for safety assessments of these environments. Moreover, root exudates of crops can also change the microorganisms in soil and control the dissipation of pesticides. Banana is one of the main fruit crops in tropical and subtropical regions. Hainan Province, Yunnan Province, and Fujian Province are the main banana-producing regions in China. The main soil types for banana planting in these regions are latosol, sandy soil, and plain alluvial soil. We selected the three tropical and subtropical soils as the research objects of our study to analyse dissipation and migration of fluopyram in these typical banana planting areas and to discuss the relevant factors affecting its dissipation and migration. This new information will facilitate data and policy support for future use of fluopyram in tropical areas. Currently, the environmental behaviour of fluopyram in the tropical soils, through processes like digestion, adsorption and leaching, has not been reported.

## Results and discussion

### Method validation

**Figure 2:**
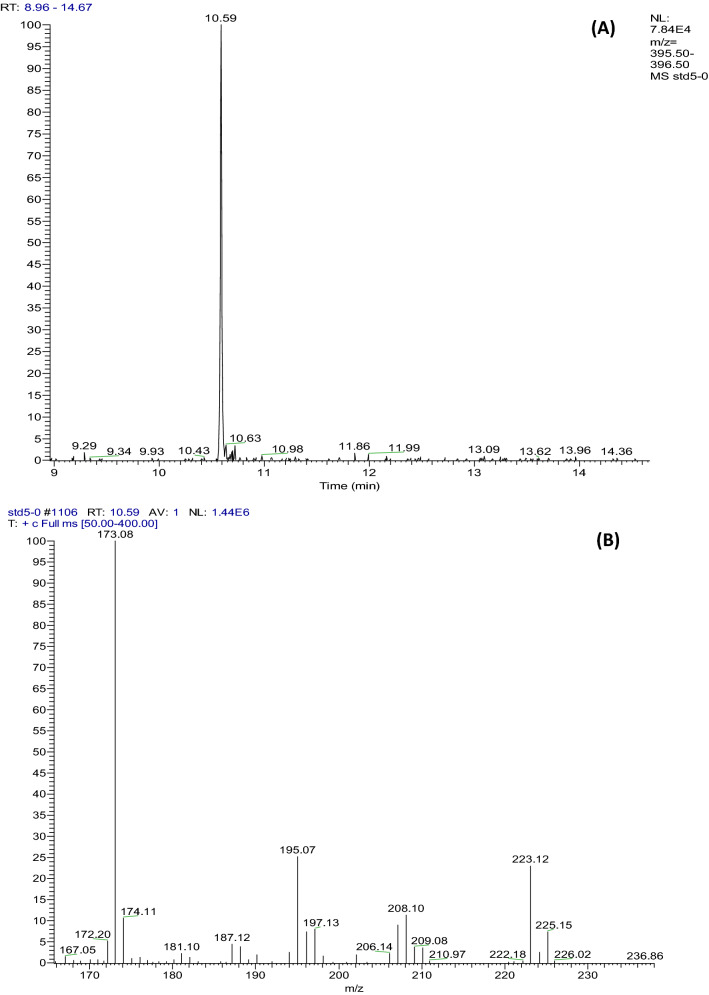
The chromatogram (**A**) and spectrogram (**B**) of fluopyram (5.0 μg/mL).

**Figure 3:**
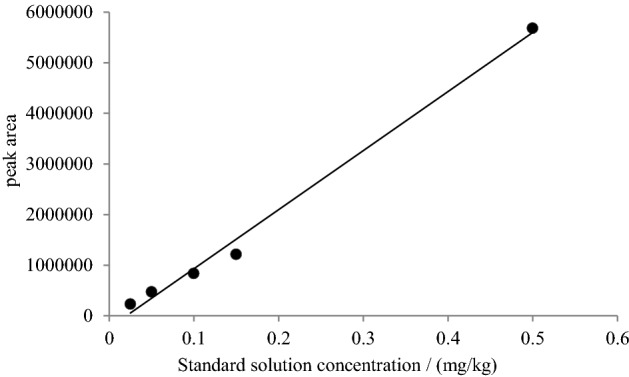
Standard curve of fluopyram solution.

Under the set operating chromatographic conditions, fluopyram was separated and eluted at a retention time of 10.59 min (Fig. 2A). The full scan mode was selected in the range *m/z* 50–500 for scanning monitoring, *m/z* 173 as the quantifier ion, and *m/z* 223 and *m/z* 195 as qualifier ions to increase the sensitivity of the target analyte (Fig. 2B). Calibration equations were calculated in 5 final concentrations (0.025, 0.05, 0.1, 0.15, 0.50 μg/mL) of fluopyram. Three sets (n = 3) were measured for each concentration, and the calibration curves were repeated. Figure 3 shows the linear regression of fluopyram, which describes the relationship between the chromatographic peak area and the pesticide concentration. The regression equation is y = − 2.37 × 10^5^ + 1.17 × 10^7^ x (R^2^ = 0.9926) and we found a good linear relationship in the concentration range of 0.025–0.50 μg/mL.

The accuracy of the method was evaluated by repeating the measurements five times for each spiked concentration. When spiked concentrations were 0.008 mg/kg, 0.600 mg/kg, and 1.000 mg/kg, the recovery rates of fluopyram in soil were in the range of 95.00–98.60%, and a relative standard deviations (RSD) were in the range of 0.99–11.70% (Table 1). According to the European Union guideline SANTE^14^, the recovery rate should be 70–120% and the RSD should be < 20%. Therefore, through the addition recovery test at three different concentration levels and the calculated RSD, the method adopted in this study can give satisfactory results with good repeatability and accuracy in terms of fluopyram test in soil. Moreover, the method meets the requirements for the pesticide residue analysis, and it is suitable for the analysis of pesticide residues of fluopyram in banana planting soil. Under the above chromatographic conditions, the limit of detection (LOD) of fluopyram in soil was 0.008 mg/kg.

**Table 1:** The recovery rate and RSD of fluopyram in soil.

Spiked level (mg/kg)	Average recovery (%)	RSD (%)
0.008	95.00	11.70
0.600	98.10	1.84
1.000	98.60	0.99

### Dynamics of chemical dissipation of fluopyram in different types of soil

The chemical dissipation behaviour of fluopyram in three banana soils was studied. We observed that its dissipative kinetics was consistent with the first-order kinetic equation C_t_ = C_0_e^−kt^ (Table 2). The dissipative kinetic equations of the analysed soils were C_t_ = 2.98e^−0.015t^, C_t_ = 3.59e^−0.019t^, and C_t_ = 2.96e^−0.012t^ for Hainan latosol, Yunnan sandy soil, and Fujian plain alluvial soil, respectively. The half-lives of the same soils were 46.21 days, 36.48 days, and 57.76 days for Hainan latosol, Yunnan sandy soil, and Fujian plain alluvial soil, respectively. Our findings indicate that fluopyram exhibited a high persistence, which is consistent with the results from Vargas-Pérez^15^. They showed that fluopyram has high persistence in greenhouse crops and could be detected 44 days after the end of the experiment. Although the degradation half-life of fluopyram in tropical soils is relatively longer, it is relatively safe for the tropical environment^16^.

**Table 2:** The chemical dissipation kinetic parameters of fluopyram in different types of soil.

Type of soil	First-order kinetic equation	Dissipation rate constant	Half-life T_1/2_/day	R^2^
Hainan latosol	C_t_ = 2.98e^−0.015t^	0.015	46.21	0.809
Yunnan sandy soil	C_t_ = 3.59e^−0.019t^	0.019	36.48	0.912
Fujian plain alluvial soil	C_t_ = 2.96e^−0.012t^	0.012	57.76	0.745

**Figure 4:**
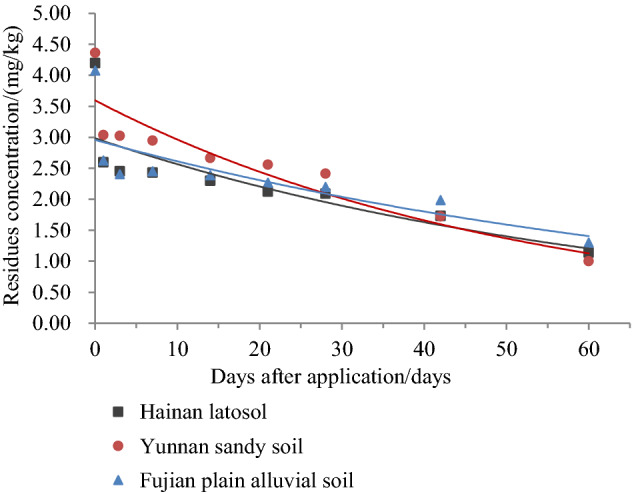
The dissipation trends of fluopyram in different banana planting soils.

In our experiment, Hainan latosol, Yunnan sandy soil, and Fujian plain alluvial soil were selected for analysis, and their physical and chemical properties are shown in Table 3. Under the same conditions, the chemical dissipation rate of fluopyram varied depending on the soil type (from the fastest to the slowest in the descending order: Yunnan sandy soil, Hainan latosol, Fujian plain alluvial soil). The dynamic dissipation curve is shown in Fig. 4. Previous studies have reported that the dissipation rates of pesticides in soil are closely related to factors such as soil type, pH, organic matter content, cation exchange capacity, and microorganisms^17–20^. Felsot and Dzantor^21^ found that the co-metabolism of high concentrations of pesticides in soil can be enhanced by increasing the content of organic matter to stimulate the biological activity of microorganisms. This yields an increase in the pesticide dissipation rate. In our experimental study, the dissipation rate of fluopyram in Yunnan sandy soil was the fastest, seemingly due to the higher pH and organic matter content of this soil type. Soil microorganisms can enhance the pesticide degradation rate. Organic matter can provide energy for microorganisms in the soil^22^. The high content of soil organic matter is conducive to the reproduction of microorganisms, affecting the number and activity of the microorganisms, which influence the degradation of pesticides. Moreover, large voids were observed in the plain alluvial soil, which was usually permeable to air and water, and it facilitated cation exchange. However, its water retention and nutrient storage capacity were very low. At the same time, it is not conducive to the retention of microorganisms, making it ineffective for the dissipation of pesticides^23^.

**Table 3:** The physical and chemical properties of tested soil.

Type of soil	pH	Content of organic matter (%)	CEC (cmol/1000 g)	Water content (%)
Hainan latosol	5.40	1.54	9.90	2.08
Yunnan sandy soil	7.50	2.42	13.33	1.00
Fujian plain alluvial soil	5.09	1.46	23.01	0.38

### Adsorption of fluopyram in three soils

**Figure 5:**
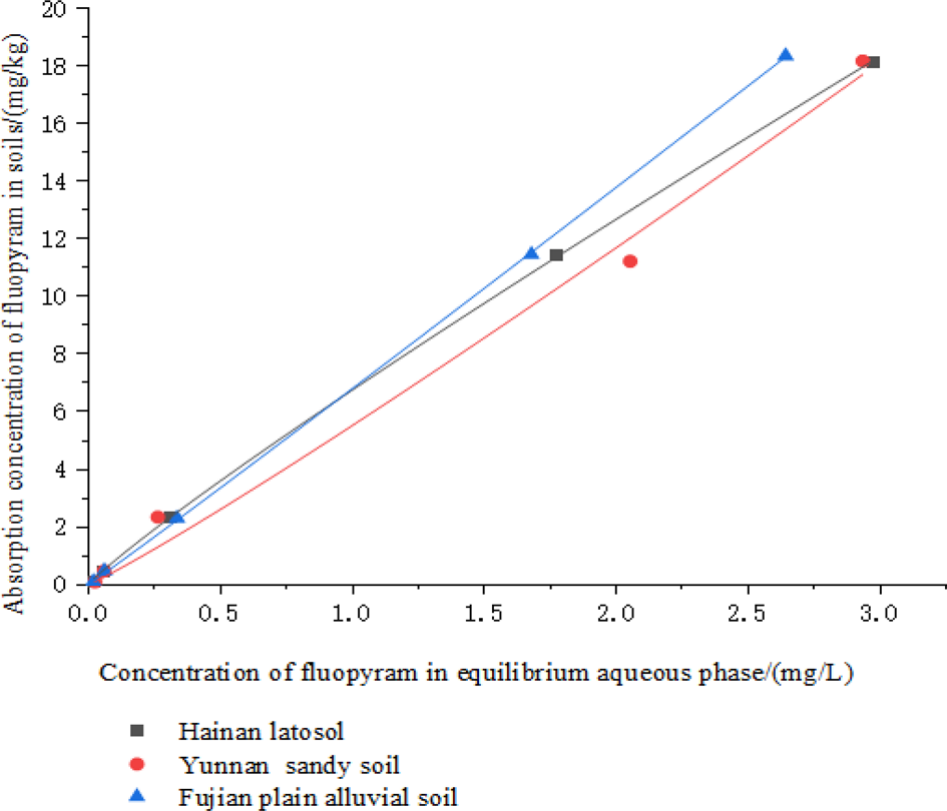
The adsorption isotherms of fluopyram in three types of soil.

The adsorption parameters of fluopyram in the three banana cultivation soils are listed in Table 4. We found that the adsorption of fluopyram in the three soils was in accordance with the Freundlich adsorption equation C_s_ = K_f_ × C_e_^1/n^, and all R^2^ values were > 0.99. Therefore, the fitting description of the Freundlich adsorption isotherm is more reasonable. The organic carbon adsorption coefficient *K*_*oc*_ is usually used to express the mobility of pesticides in soil, and the free energy (Δ*G*) was used to determine the mechanism and degree of pesticide adsorption. Table 4 and Fig. 5 show that the adsorption properties of fluopyram in different banana cultivation soils were different. The *K*_*oc*_ value of fluopyram in the three banana soils was > 2 × 10^4^, so fluopyram was easily adsorbed in the soil. The Fujian plain alluvial soil had the highest adsorption capacity, followed by the Hainan latosol; the Yunnan sandy soil had the lowest adsorption capacity. The adsorption free energy values (Δ*G*) for fluopyram in all the three soils were negative (< 40 kJ/mol), indicating that the adsorption process of fluopyram in soil was spontaneous and manifested as physical adsorption.

**Table 4:** The adsorption parameters of fluopyram in different planting soils.

Type of soil	Freundlich equation	K_d,F_ (L/kg)	1/n	R^2^	Kom (L/kg)	ΔG (kJ/mol)
Hainan latosol	C_s_ = 6.75 × C_e_^0.91^	6.75	0.91	0.999	43,636.36	− 24.26
Yunnan sandy soil	C_s_ = 5.52 × C_e_^1.08^	5.52	1.08	0.992	22,809.92	− 22.79
Fujian plain alluvial soil	C_s_ = 6.8 × C_e_^1.02^	6.80	1.02	0.999	46,735.40	− 24.42

The migration ability of pesticides in the soil is associated with their adsorption in the soil^24^ and the strength of the adsorption of pesticides mainly depends on the properties of the pesticide and the nature of the soil^25,26^. The lower the water solubility of the pesticide, the higher is its adsorption in the soil. The solubility of fluopyram in pure water was 0.016 g/L (20 °C), which indicates that it was hardly soluble in water, so the easy adsorption of fluopyram in soil mostly depended on its water solubility. At the same time, the adsorption capacity of the soil for pesticides is related to the organic carbon adsorption coefficient *K*_*oc*_. The larger the *K*_*oc*_ value, the higher is the adsorption capacity. In our experiment, the *K*_*oc*_ value analysis showed that the effect of fluopyram in the three banana soils was as shown further. The adsorption capacity descended in the order: Fujian plain alluvial soil > Hainan latosol > Yunnan sandy soil. The *K*_*oc*_ value is related to the physical and chemical properties of soils (pH and organic matter content)^27,28^_._ The adsorption of pesticides in soil decreases with an increase in soil pH within a certain range. The experimental results of Kumar showed that in the pH range of 6–8, the adsorption quantity decreased with the increase in pH, and the adsorbability became weaker gradually^29^. As the main form of pesticide adsorption in soil is the molecular state and its anions are difficult to get adsorbed on soil^30^. Fluopyram dissociates in soil. The pH increased, the dissociation of fluopyram into ions increased, and the adsorbability reduced. This also explains the negative correlation between the organic carbon adsorption coefficient and pH. Table 3 shows that the pH and organic matter content of the Fujian plain alluvial soil were the lowest, so fluopyram had the highest adsorption capacity in this type of soil.

### Leaching and migration of fluopyram in three soil columns

The fluopyram content distribution in different soil leaching columns is shown in Table 5. We found that under the same conditions, fluopyram was not detected in the leaching water of the three types of soil columns. Most of them were found in in the top 0–10 cm of soil column, a small amount was found at a 10–20 cm depth, and only a little or none found at soils depths of 20–30 cm. The movement of fluopyram in the different soil pillars was different. With the extension of the soil columns, the fluopyram content in the Hainan latosol and Fujian plain alluvial soil gradually decreased; the content of fluopyram in Yunnan sandy soil first increased and then decreased, with the highest content at 5–10 cm. We note that fluopyram had the highest leachability in Yunnan sandy soil, followed by Hainan latosol, it was the lowest in Fujian plain alluvial soil. The pattern of adsorption of fluopyram in soil was the opposite. As the fluopyram content in the 0–10 cm section of the leaching soil column was far greater than 50% of the total amount of pesticide, fluopyram had very low leachability and migration in the three banana soils, and all of them were difficult to leach.

**Table 5:** The content distribution of fluopyram in different soil columns.

Type of soil	R_1_ (μg)		R_2_ (μg)	R_3_ (μg)	R_4_ (μg)
	0–5 cm	5–10 cm	10–20 cm	20–30 cm	Shower water
Hainan latosol	52.64	28.64	21.69	0.99	NF
Yunnan sandy soil	18.69	48.00	32.91	1.80	0.33
Fujian plain alluvial soil	51.18	38.04	10.61	NF	NF

Pesticide leaching is an important factor in groundwater pollution^31^. The higher the leachability of pesticides, the greater the possibility of it entering groundwater. In our experiment, most of the fluopyram remained within depths of 0–5 cm and 5–10 cm in the three soil columns. The sum of pesticide residues in these two soil layers accounted for 81.48%, 66.69%, and 89.22% of the amount of pesticide applied in the three soils, respectively. The leaching of fluopyram in the three banana soils was poor due to the low water solubility of fluopyram and the high adsorption of fluopyram to the soil. The higher the soil adsorption capacity, the higher the tendency of fluopyram in the soil to be enriched in the solid phase, and thus, the leaching of pesticides in the soil gets considerably reduced^32^. Although fluopyram was mainly concentrated in the 0–10 cm section in the Yunnan column, the pesticide residue in the 10–20 cm section was also relatively large, accounting for 32.91% of the total applied amount, and it moved to the leaching water. The experimental results showed that the pesticide had the highest leachability in the sandy soil. Since the fluopyram acidity coefficient (pKa) was 13.34 ± 0.46 (weakly acidic), the pH value of the soil affected its degree of ionisation and hydrolysis in the soil^30,33^. Thus, the leachability of fluopyram in the weakly alkaline sandy soil was higher than that in the acidic latosol and the plain alluvial soil.

## Conclusion

It is important to study the environmental behaviour of fluopyram in tropical soil to understand its properties and ensure the safety of its application for banana cultivation. In this study, the dissipation, adsorption, and leaching dynamics of fluopyram in different typical banana planting soils were examined. The results showed that the dissipation of fluopyram and was the fastest in Yunnan sandy soil, its dissipation half-lives in Hainan latosol, Yunnan sandy soil and Fujian plain alluvial soil were 46.21 days, 36.48 days, and 57.76 days, respectively, which were longer than those of fluopyram in the Shandong and Anhui soils^12^. This may be explained by the fact that the banana planting areas in Hainan, Yunnan, and Fujian belong to tropical or subtropical areas, where the pH value and organic matter content of the soils are relatively low. Meanwhile, crop root exudates and field and experimental soil environment affected the soil microorganisms, thus influencing the dissipation rate of fluopyram. In this experiment, the *K*_*f*_ values of all the three tested soils were > 2, indicating that fluopyram in banana soil had low migration risk, and it could not penetrate the deeper parts of soil to pollute the underlying groundwater^24^. Due to the low solubility of fluopyram in water, it was easily adsorbed by the soil and had poor leaching. However, the adsorption properties of fluopyram differed in the three soils, and the highest adsorption was observed in Fujian plain alluvial soil due to the influence of soil pH and organic matter content. The leaching property of the pesticide was different in each of the three soils, with the highest leaching properties in the Yunnan sandy soil. Although fluopyram does not easily migrate in banana planting soil, it has good systemic properties, high residue in surface soil, and relatively long half-life, which will increase its accumulation in surface soil and affect subsequent crops.

## Materials and methods

### Chemicals and reagents

The fluopyram standard was purchased from the Environmental Protection Monitoring Institute of the Ministry of Agriculture of China at a concentration of 1000 mg/L. Analytical grade acetonitrile, acetone, dichloromethane, and sodium chloride were purchased from the Guangzhou Chemical Reagent Factory. Chromatographic grade Methanol and n-hexane were available from Thermo Fisher Scientific. Purified water was prepared using a Milli-Q reverse osmosis system (Millipore, Milford, MA, USA). Strata Florisil (FL-PR) 500 mg/6 mL SPE manufactured by Strata™ (5.0 mL n-hexane–acetone (9:1, V/V) solution pre-rinsing cartridge).

A standard solution of 1000 μg/mL fluopyram was diluted in n-hexane, and the matrix extract of the blank sample was obtained by the extraction method. The matrix standard solutions of 0.025, 0.05, 0.10, 0.15 and 0.50 μg/mL were obtained by the step dilution. All prepared solutions were stored at temperature of 4 °C until further use.

### Soil sample collection

Hainan latosol was collected from the Bailian banana experimental base in Chengmai (Hainan), Yunnan sandy soil was collected from Taoyuan banana experimental base in Longtou Street, Kunming (Yunnan) and Fujian plain alluvial soil was collected from the Zhangzhou banana experimental base (Fujian). 5–10 soil sampling points were randomly selected in each banana experimental base; the soil samples were collected from depths of 0–10 cm, and debris such as gravel, weeds, and plant roots were removed from each sample. The soil samples were obtained by the quarter method after mixing, dried, and stored after 20 mesh screening.

### Extraction and purification of flupyram

Soil sample extraction was conducted as follows: in a 200 mL conical flask, 20.0 g of the drying soil sample and 40.0 mL acetonitrile was added. After shaking on a reciprocating shaker for 2 h, the mixture was filtered through filter paper. The filtrate was transferred to a stoppered measuring cylinder with 6.0 g NaCl. The stopper was inserted, and the mixture was vigorously shaken for 2 min. The mixture was left at 25 ± 2 °C for more than 30 min to separate the acetonitrile and aqueous solutions. Meanwhile, 10.0 mL of the supernatant were accurately transferred into a 100 mL round-bottom flask and concentrated by a rotatory evaporator at 40 °C to near dryness, which was dissolved in a 5.0 mL n-hexane–acetone (9:1, v/v) solution, vortexed, and mixed well for purification.

Water sample extraction is shown below. A 20 mL water sample was transferred to a separatory funnel with 40.0 mL dichloromethane. After vigorously shaking it for 2 min and then letting it stand for 30 min, the lower layer solution was collected in a 100 mL round-bottom flask. The collected fluid was concentrated by a rotatory evaporator at 40 °C to near dryness and dissolved in 5.0 mL n-hexane–acetone (9:1, v/v) solution, vortexed, and mixed well for purification.

Sample purification is described below. A 5.0 mL n-hexane–acetone (9:1, v/v) was used to preach the Strata Florisil (FL-PR) 500 mg/6 mL extraction column. When the leaching solvent level reached the surface of the column adsorption layer, the solution sample was immediately poured into the column be purified. Then, the purified solution was collected in a 100 mL round-bottom flask. A 5.0 mL *n*-hexane–acetone (9:1, v/v) solution was used to rinse the round-bottom flask residuum, after which the rinse solution was applied to elute the Florisil column. The rinsing and elution steps were repeated three times. The collected fluid was concentrated by a rotatory evaporator at 40 °C to near dryness and dissolved in 2.5 mL *n*-hexane for analysis.

### Instrumental condition

The test was performed using the Theomer DSQII gas chromatography-mass spectrometer (GC–MS) with Xcalibur 2.0, software for data acquisition and analysis. A SLB-5MS analytical column (30 m × 0.25 mm × 0.25 μm) was used as chromatographic column. The injection volume was 1 μL without split injection, the carrier gas was helium (He, ≥ 99.999% purity), and the carrier gas flow rate was set to 1.0 mL/min. The protective gas was nitrogen (N_2_, ≥ 99.999% purity), and the injection port temperature was 250 °C. The chromatographic column temperature program was set as follows: the initial temperature at 80 °C was maintained for 1 min; then it was raised to 240 °C at a speed of 20 °C/min and maintained for 3 min; finally, the temperature was raised at a rate of 50 °C/min until 280 °C, where it was maintained for 7 min.

The MS was operated in electron ionisation (EI) mode with an ionising energy of 70 eV. MS data were acquired in both full scan (*m/z* 50–500) mode for identification and selected ion monitoring (SIM) mode for quantification. The temperatures of the ion source and transfer line were 250 °C and 280 °C, respectively. The retention time of fluopyram was 10.59 min. The quantifier ions were *m/z* 223, and the qualifier ions were *m/z* 195 and *m/z* 173.

### Analytical method validation

First, we addressed the linearity. The matrix standard of fluopyram was prepared in the range of 0.025–0.50 μg/mL and the determination was carried out, with the concentration of fluopyram matrix standard solution as the abscissa and the peak area obtained from the GC–MS as the ordinate. Linearity was calculated by plotting the relationship between the concentration and the peak area.

The sensitivity analysis relied on the LOD and the limit of quantitation (LOQ). To evaluate the sensitivity of the method, they were obtained by adding the standard solution of fluopyram at the lowest concentration level in line with the requirements of the analytical method for blank samples. The LOD was the corresponding concentration when the signal-to-noise ratio (S/N) was 3, and S/N = 10 corresponds to the LOQ.

Accuracy and precision were estimated as well. To determine the reliability of the method, fluopyram standard solutions with different concentrations were added to the blank sample for the recovery experiment. Fluopyram standard solutions with concentrations of 0.008, 0.600, and 1.000 mg/kg were added to the blank samples. This procedure was repeated five times for each concentration. The samples were subjected to extract, purify and analysis under the method the same conditions as described above. The recovery was calculated for the accuracy of the method, and the RSD was calculated for the precision.

### Soil dissipation experiment

In a number of 100 mL clean and sterilized conical flasks with covers, 20.0 g of soil was added (net weight converted by water content); then, 0.1 mL 1000 μg/mL fluopyram standard solution was pipetted into the conical flasks. Ultrapure water was added. The water was controlled to occupy 60% of the total volume. The flasks were shaken on a constant temperature oscillator for 2 min to mix the fluopyram evenly. Then, they were placed in an artificial climate incubator and exposed to light at 25 ± 2 °C for 12 h per day. According to the different soil types, they were divided into three treatment groups: Hainan, Yunnan, and Fujian. Each treatment group had three parallels and three blanks. The detection intervals were 2 h, 1, 3, 7, 14, 21, 28, 42 and 60 day, while the detection of fluopyram was performed based on the interval according to the shown methods. The dissipation kinetics of fluopyram in banana planting soil conformed to the first-order kinetic equation C_t_ = C_0_e^−kt^, where *C*_*t*_ is a pesticide concentration (mg/kg) at different times (day), *C*_0_ is an initial concentration (mg/kg), and *k* is the dissipation rate constant. The half-life of fluopyram is determined using Eq. (1).

1 $$T_{1/2} = \, \ln 2/k$$

### Soil adsorption experiment

Using the oscillation balance method, 5.0 g of soil was put into the 250 mL conical flasks with cover, which contained 25 mL fluopyram aqueous solutions with mass concentrations of 0.02, 0.1, 0.5, 2.5 and 4.0 mg/L (containing 0.01 mol/L CaCl_2_), respectively. The soils were divided into three treatment groups: Hainan, Yunnan, and Fujian (based on the different soil types). The fluopyram aqueous solution and the blank soil aqueous solution (both containing 0.01 mol/L CaCl_2_) were used as controls. Each treatment group had three replicates. The conical flasks were then placed in a constant temperature oscillator at 25 ± 2 °C for 24 h to prepare the suspension. The suspension was transferred to a centrifuge tube for high-speed centrifugation, and 80% of the total volume of the supernatant was used for determination. The fluopyram in the supernatant was extracted and determined under the methods as described above, and the Freundlich equation model (see Eq. 2) was used to describe the adsorption law for fluopyram in soil.

2 $${\text{Freundlich: }}C_{s} = K_{f} \times C_{e}^{1/n}$$

where *C*_*s*_ is adsorption content of pesticide in soil (mg/kg), *C*_*e*_ is concentration of the pesticide in aqueous solution at adsorption equilibrium (mg/L), *K*_*f*_ is the soil adsorption coefficient of the Freundlich model (L/kg), indicating the pesticide adsorption capacity of the soil and 1/*n* is a slope rate of the curve between *C*_*s*_ and *C*_*e*_, reflecting the heterogeneity of the adsorbent surface.

The relationship between the adsorption free energy of soil to pesticides (Δ*G*, kJ/mol) and the soil adsorption coefficient *K*_*oc*_ is expressed using Eq. (3).

3 $$\Delta G \, = - RT\ln K_{oc}$$

where *K*_*oc*_ is the soil adsorption coefficient (K_oc_ = K_f_/OC × 100) expressed by organic carbon content (L/kg), *OC* is soil organic carbon content (%), *R* is the molar gas constant (J/K mol), and *T* is absolute temperature (K).

### Soil leaching experiment

A plexiglass tube with an inner diameter of 5 cm and a length of 40 cm was used as a packed column. A layer of cotton, a 1 cm thick quartz sand layer, and a layer of filter paper were added at the bottom of the column. Dry soil (700–800.0 g) was weighed for filling, and the column was fully wetted with ultrapure water to prepare a 30 ± 0.2 cm high leaching soil column. 0.1 mL of 1000 μg/mL fluopyram solution was further added to 5.0 g of soil. After the solution completely volatilized, it was evenly spread on the top of the soil column, and a layer of filter paper and a layer of 1 cm thick quartz sand were added to the top of the soil. During the test, ultrapure water was used for washing the soil column for 10 h at a speed of 30 mL/h, and the leaching solution was collected. After washing, the soil column was removed and was cut into four sections of 1–5, 5–10, 10–20 and 20–30 cm. The residues of fluopyram in the soil samples and leaching solutions were extracted and determined under the methods as described above. According to the three soil types, they were divided into Hainan, Yunnan and Fujian treatment groups, where each group received another parallel treatment.
